# Initial development and testing of an exhaled microRNA detection strategy for lung cancer case–control discrimination

**DOI:** 10.1038/s41598-023-33698-8

**Published:** 2023-04-24

**Authors:** Miao Shi, Weiguo Han, Olivier Loudig, Chirag D. Shah, Jay B. Dobkin, Steven Keller, Ali Sadoughi, Changcheng Zhu, Robert E. Siegel, Maria Katherine Fernandez, Lizett DeLaRosa, Dhruv Patel, Aditi Desai, Taha Siddiqui, Saurabh Gombar, Yousin Suh, Tao Wang, H. Dean Hosgood, Kith Pradhan, Kenny Ye, Simon D. Spivack

**Affiliations:** 1 Pulmonary Medicine, Albert Einstein College of Medicine, Bronx, NY USA; 2 Pharmacology and Toxicology, College of Pharmacy, University of Arizona, Tucson, AZ USA; 3 Center for Discovery and Innovation, Nutley, NJ USA; 4 Merk, Kenilworth, NJ USA; 5 Pathology, Albert Einstein College of Medicine, Bronx, NY USA; 6 Pulmonary Medicine, Icahn School of Medicine at Mount Sinai, James J. Peters Veterans Affairs Medical Center, New York, USA; 7 Montefiore Health System, Bronx, NY USA; 8 Genetics, Albert Einstein College of Medicine, Bronx, NY USA; 9 Reproductive Sciences (in Obstetrics and Gynecology), Columbia University, New York, USA; 10 Genetics and Development, Columbia University, New York, USA; 11 Biostatistics, Albert Einstein College of Medicine, Bronx, NY USA; 12 Epidemiology, Albert Einstein College of Medicine, Bronx, NY USA; 13 Systems and Computational Biology, Albert Einstein College of Medicine, Bronx, NY USA

**Keywords:** Biotechnology, Cancer, Biomarkers

## Abstract

For detecting field carcinogenesis non-invasively, early technical development and case–control testing of exhaled breath condensate microRNAs was performed. In design, human lung tissue microRNA-seq discovery was reconciled with TCGA and published tumor-discriminant microRNAs, yielding a panel of 24 upregulated microRNAs. The airway origin of exhaled microRNAs was topographically “fingerprinted”, using paired EBC, upper and lower airway donor sample sets. A clinic-based case–control study (166 NSCLC cases, 185 controls) was interrogated with the microRNA panel by qualitative RT-PCR. Data were analyzed by logistic regression (LR), and by random-forest (RF) models. Feasibility testing of exhaled microRNA detection, including optimized whole EBC extraction, and RT and qualitative PCR method evaluation, was performed. For sensitivity in this low template setting, intercalating dye-based URT-PCR was superior to fluorescent probe-based PCR (TaqMan). In application, adjusted logistic regression models identified exhaled miR-21, 33b, 212 as overall case–control discriminant. RF analysis of combined clinical + microRNA models showed modest added discrimination capacity (1.1–2.5%) beyond clinical models alone: all subjects 1.1% (*p* = 8.7e−04)); former smokers 2.5% (*p* = 3.6e−05); early stage 1.2% (*p* = 9.0e−03), yielding combined ROC AUC ranging from 0.74 to 0.83. We conclude that exhaled microRNAs are qualitatively measureable, reflect in part lower airway signatures; and when further refined/quantitated, can potentially help to improve lung cancer risk assessment.

## Introduction

Non-invasive access to the lung is a major challenge, but is plausibly addressed because of the anatomic continuity between ambient air, bronchial airways, and the deep lung alveolar epithelium. Coupled to this anatomic opportunity is a clinical exigency, that lung disease detection and monitoring could be improved by molecular biomarkers. In the lung cancer early diagnostics arena, there is a consensus that the positive predictive value, efficiency, and mortality benefit of low dose CT screening modalities for early lung cancer detection, clinical diagnostics, or prevention strategies, could be better leveraged by defining up-front an even higher risk subpopulation^1–5^. That is, clinical risk factors of age, smoking status, and tobacco dose, when combined into sophisticated risk models^6,7^, still do not adequately capture overall risk nor define the highest risk subgroups, as most of the risk for lung cancer remains unexplained by standard clinical factor-based risk profiling^2,8^. Therefore, the pursuit of molecular markers of risk is pivotal to improving current lung cancer screening and prevention efforts^1,2,8,9^, to focus on those individuals most likely to benefit. Blood-based markers have been suggested^10,11^. Assessments of the broad epithelial field for messenger RNAs in bronchial brushings have been convincing^12,13^, and that for microRNAs suggestive^14^. The transcriptomes of the main lung cancer tumor histologies have been published^15,16^. Non-invasive molecular risk-assessment tools for exhaled microRNAs are rarely reported^17–20^, have not been rigorously evaluated or quantitated, and are not in clinical use at present.

This initial report describes the development of an exhaled microRNA approach to non-invasive interrogation of the lung that is both lower airway-derived, and population-applicable, for the purpose of developing a lung cancer risk biomarker. As first steps, we confirmed the technical feasibility of detecting microRNAs in exhaled breath condensate (EBC). MicroRNA isolation from unfractionated whole EBC was optimized. Our reverse transcription (Universal-tagged RT)^21–23^ platform was adapted, and showed enhanced sensitivity/performance of the coupled URT-PCR platform in EBC, as compared to a standard, widely used probe-based RT-PCR platform (TaqMan, Invitrogen)^24–26^. MicroRNA URT and PCR steps were then further optimized. A candidate microRNA pool was then generated, including upregulated microRNAs that differentiated homogenized human lung NSCLC tumors versus paired remote non-tumor tissue from the same individual surgical resections, using RNAseq from 32 surgical sample sets (ENA accession: PRJEB52036). These were then verified against analogous data from the TCGA^15,16^*.* Additional microRNAs of interest were selected from the published lung cancer literature^27–30^. Using this 24-microRNA panel, exhaled microRNA-qualitative PCR performance in discriminating those with non-small cell lung cancer (cases), and those without lung cancer (controls) drawn from similar clinical populations of individuals destined for bronchoscopy or lung resection surgery was then assessed. Starting with a robust base clinical model, for the three primary data analyses (all clinical categories, former smokers, or early stage), the exhaled qualitative microRNA-PCR biomarker data yield a modest increment in case–control discrimination, over and above clinical factor models alone.

## Materials and methods

### MicroRNA specificity testing of newly designed microRNA PCR primers

All microRNA primers were initially tested for microRNA specificity on RNA extracts from lung cell lysates, and on pilot EBC samples, using both realtime RT-qPCR and gel electropheresis with key controls. This testing included a: (a) no-RT step (to exclude gDNA-derived signal); (b) no polyA-step (to exclude messenger RNA -derived signal); (c) genomic DNA spike-in (to exclude gDNA-derived signal); and (d) water-no template blank (to exclude cDNA or PCR-product contaminated reagents). Cell culture samples were used as positive controls for EBC microRNA-PCR development: For positive controls in microRNA-PCR assay development, total RNA extracts from a set of pooled cell lines including NHBE, HBEC, A549, Hela, HTB-119 and CRL-1995 was combined and RNA extracted in conventional column (RNeasy, Qiagen). This provided a stock solution of total RNA for initial testing of microRNA-specific primers.

### EBC sample collection

**Figure 1:**
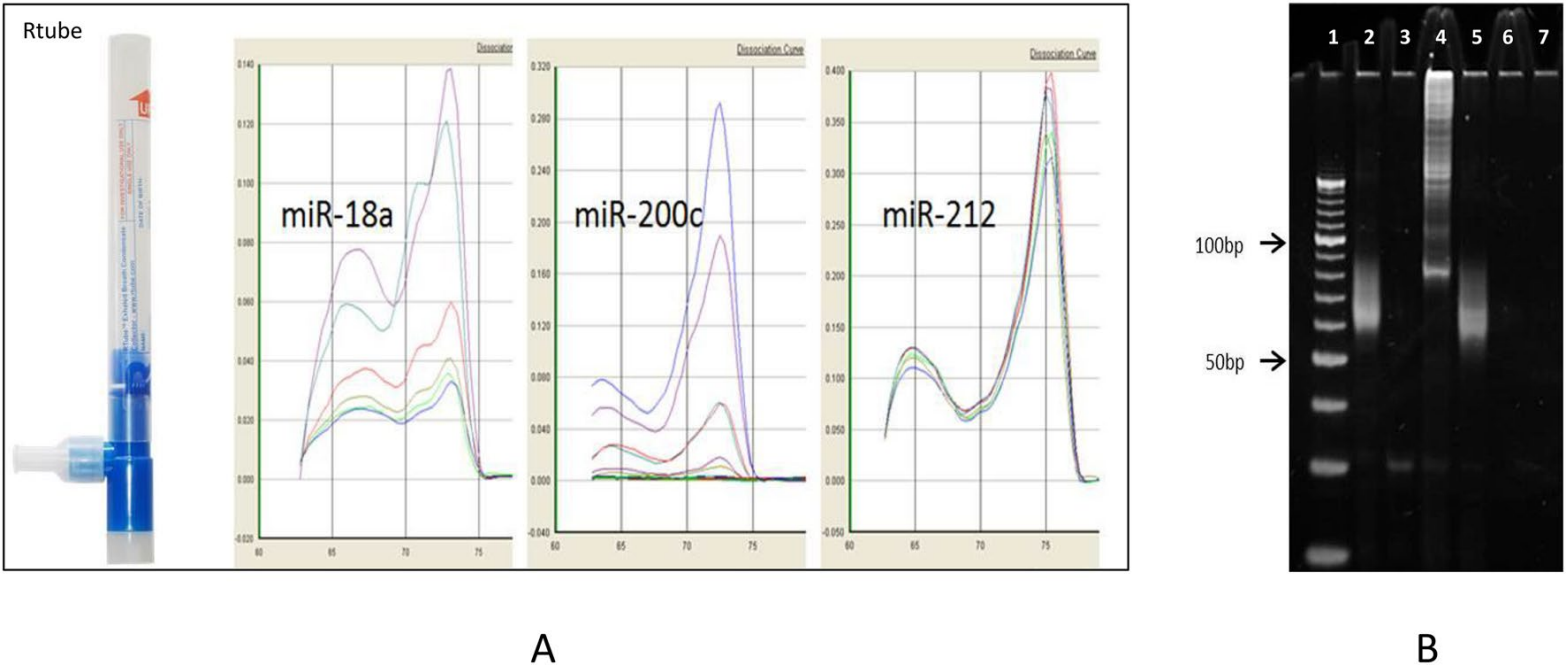
Schematic of EBC-microRNA detection and verification: (**A**, left panels) Exhaled microRNA collection and amplification. The method used the handheld disposable Rtube, typical examples of URT-PCR melt curves for miRs 18a, 200c, 212 are shown. (**B**, right gel panel): Specificity testing of designed microRNA PCR primers. PCR products were loaded in EtBr gel. Note that 40 bp URTtag + 22 bp microRNA template = 62 bp PCR product. Lane 1: 10 bp DNA ladder; lane 2: cDNA of cultured lung cell microRNA and mRNA (total RNA extract), with both polyadenylation and RT steps; lane 3: No RT step, total RNA from cultured lung cells; lane 4: cDNA of mRNA (no poly-adenylation step, total RNA from cultured lung cells); lane 5: cDNA of EBC microRNA and mRNA (all steps included); lane 6: Genomic DNA spike-in as only template; lane 7: Water blank/no template control. For a microRNA-specific PCR primer set, microRNA-size PCR bands are uniquely seen in lane 2 (cultured lung cell mix RNA extract template, all polyadenylation and RT steps included), and lane 5 (EBC RNA extract template, all polyadenylation and RT steps included). PCR product size is ~ 60–63, reflecting the 20–23 bp microRNA template, and the 40 bp URT tag integrated at the RT step^21–23^.

The EBC collection followed the recommendations of the American Thoracic Society/European Respiratory Society Task Force on EBC^31^. The RTube device (Respiratory Research, Inc) was used to collect patient’s Exhaled Breath condensate (EBC), per standard protocol (Fig. 1A). The essential features of the simple handheld RTube device are: (i) One way inhalation/exhalation valve; (ii) Small port for exhaled breath creating turbulence to impact cooling chamber walls; (iii) Exhalation cooling chamber, polypropylene with pre-cooled aluminum sleeve; (iv) Manually operated piston for capture of condensate from the RTube after collection. Briefly, before any clinically-indicated lung procedure, seated subjects were equipped with RTube /mouthpiece/noseclips and performed quiet, tidal volume breathing plus one deeper breath (sigh) per minute, collected over a 10–15 min span. Saliva was to be swallowed, and excess saliva was trapped by RTube device by design. Any coughing was instructed to be done off of the mouthpiece, to minimize oral contamination of the EBC specimen. A bare minimum of 100 ul of EBC was the goal, and achieved in > 75% of subjects. Over 50% of individuals collected > 500ul EBC.

### Optimization of EBC microRNA extraction

Optimization of EBC microRNA extraction was performed, comparing ethanol alone, trizol alone, speed vacuum alone, column-based method alone (Supplemental Figure S1A) and combinations of the above with glycogen and carrier RNA (Supplemental Figure S1B). Optimal was ethanol precipitation with glycogen carrier molecule, and the final protocol included a biochemical/Trizol-based isolation. *EBC RNA extraction protocol detail:* For total RNA extraction, whole unfractionated EBC was concentrated by ethanol precipitation and then purified by Trizol (Invitrogen) per manufacturer protocol and lab optimized protocol. The following components were added into a capped polypropylene tube and thoroughly mixed, including 100–400 ul of EBC sample, 40 ul of 3 M sodium acetate (pH 5.5), 5 ul of 5 ug/ul glycogen carrier, and 1100 ul of 100% cold ethanol. The mixture was chilled at − 80 °C for 30 min and then centrifuged at 14,000 rpm for 20 min at 4 °C. Then, the supernatant was discarded and the pellet was rinsed with cold 70% ethanol twice, and air-dried. The pellet was then dissolved in 0.5 ml of Trizol. Total RNA was purified per the Trizol manufacturer protocol. The RNA pellet was dissolved in 15 ul of RNase-free water.

### Universal RT primer optimization for microRNAs

For microRNAs, the big issue of universal RT primer is that it can often amplify similar size products from both cDNA with polyadenylation and cDNA without polyadenylation. Every microRNA primerset will be tested on two different templates (cell cDNA with polyadenylation and cell cDNA without polyadenylation). The microRNA primers sequences used in the study are listed in Supplemental Table S2.

### EBC MicroRNA PCR analysis

The overall strategy was to amplify mature microRNAs by the previously published URT-PCR lab protocol^21–23^, involving poly-A tailing using a one-base anchored and universal tagged oligo-dT-RT strategy, and a microRNA-specific forward primer coupled to a universal, human-unique tag-specific reverse primer, in aggregate precluding false gDNA amplification. Individual steps and details follow.

Poly(A) Tailing: The Poly(A) Tailing Kit (Ambion) was used to polyadenylate the 3' termini of microRNA. First, ATP was diluted to 1% of the original concentration. Then, the following components were added into a PCR tube and thoroughly mixed, including 2 ul of 5 × buffer, 0.8 ul of MnCl_2_ (25 mM), 0.4 ul of diluted ATP, 0.25 ul of enzyme and 6.55 ul of total RNA from EBC. The mixture was incubated at 37 °C for 30 min.

Reverse transcription: Reverse transcription was performed with 10 µl of the *E. coli* Poly(A) Polymerase (*E*-PAP) treated total RNA using Superscript III reverse transcriptase (Invitrogen) as follows. RNA template was added to a master mix containing 1 µl of 100 µM universal oligo-dT-adapted universal URT primer^23^*,* 1 µl of dNTP mix (each base 10 µM) and 1 µl of DNase/RNase-free water. Total volume was adjusted to 13 µl with DNase/RNase-free water. The solution was incubated at 65   C for 5 min and then cooled on ice. A master mix containing 4 µl of 5X first-strand buffer, 1 µl of 0.1 mM DTT, 1 µl RNaseOUT (Invitrogen) and 1 µl SuperScript III per RT sample was prepared and added to each sample. The samples were incubated at 42  C for 30 min, 50  C for 30 min, followed by 70  C for 15 min.

Realtime PCR: Typically, the RT reaction was diluted 1:20 and 2 µl used in the realtime PCR of microRNAs with the microRNA transcript-specific forward PCR primers (Supplemental Table S2, n = 25 primersets) and a matched (tag-directed) reverse primer^21–23^. cDNA template was added to a master mix containing 10 ul of 2 × PowerSYBR green master mix (Applied Biosystem), 1 ul of 10 uM primers mix and 7 ul of DNase/RNase-free water. The reaction was incubated in an Applied Biosystems 7500 real-time PCR system at 95 °C for 10 min, followed by 45 cycles of 95 °C for 15 s, 60 °C for 15 s and 72 °C for 32 s. After that, dissociation stage/melting curve analysis was performed. In developing each primerset, primers were designed to produce a single unique melting curve on known microRNA extracts from lung cell lines. Multiple separate positive and negative controls in both cell lines and EBC sample standards were run, including (a) gDNA spike (to exclude false gDNA amplification) (b) no-RTase (to further exclude false gDNA amplification); (c) no polyadenylation (to exclude false messenger RNA amplification); (d) no template (to exclude reagent contamination by PCR product).

### Data cleaning/scoring

Since microRNAs are all of near-identical size, it was left to base composition/melting temperature to be the major distinguishing feature between individual microRNAs. The criteria for including or excluding a micro-RNA-derived PCR product as present or absent was dependent on the data from the melting curves. If a sample had the same melting temperature (Tm) as the positive control from cell lines for that microRNA primerset, and the Ct value was less than 44, it was called “positive”. If a reaction sample had no visible melting curve, or the visible melting curve displayed greater than + /− 1.5 °C different Tm from the melting curve from the positive, individual miR-specific control from cell lines, it was called “negative”. We chose one convention for overall scoring of samples – at least one of two replicates must be positive.

The housekeeper control chosen, based on literature, and the most ubiquitous presence in our EBC samples, was miR-423-3p^32,33^*.* From previously described studies^34–36^*,* hsa-miR-16, hsa-miR-26b, hsa-miR-92, hsa-miR-423, hsa-miR-374, have been used as housekeeper controls. However, we found suboptimal housekeeper miR repeatability/precision by delta CT values > 2.0 on technical replicate. This precluded consistently accurate quantitation of candidate housekeeper microRNAs (hsa-miR 423-5p, others), in many instances, by either URT-PCR or TaqMan-PCR. This precluded sufficiently reliable target microRNA.quantitation in EBC using these methods and platforms. Therefore, only qualitative data (microRNA present/absent) were analyzed from the clinical EBC samples, despite realtime monitoring of PCR amplifications.

### URT-PCR versus Taqman PCR

In order to compare URT-PCR and Taqman PCR, let-7a, let-7f., miR-18a, miR-21, miR-26a, miR-140, miR-212, miR-423-3p, miR-708 and miR-767 were chosen as arbitrary targets (Table 1). Samples chosen were 100 ng/ul of HBEC total RNA, 1 ng/ul of HBEC total RNA and RNA of EBC. RNA of EBC was purified by the optimized miRNA extraction method in this study. URT-PCR was performed by our previously published URT-PCR lab protocol^21–23^ and Taqman PCR was performed by Invitrogen Taqman miRNA assays according to TaqMan-designed primers and the manufacturers protocol. For URT-PCR, controls included cDNA of miRNA, no RT (omitted RTase), cDNA of mRNA (no poly-adenylase), genomic DNA spike-in, and water blank (no template). MiR-423-3p was used as a housekeeper, though it was not detected by TaqMan system. During URT-PCR analysis, melting curves were examined in order to verify correct PCR products.

**Table 1:** URT-qPCR versus Taqman qPCR.

	URT-qPCR			Taqman qPCR		
Templates average Ct targets	100 ng/ul total RNA	1 ng./ul total RNA	EBC miRNAs	100 ng/ul total RNA	1 ng./ul total RNA	EBC miRNAs
let-7a	17.15	24.03	36.60*	19.53	27.99	ND
let-7f	24.64	30.48	ND	22.78	31.76	ND
miR-18a	25.30	27.38	33.46*	21.30	31.03	ND
miR-21	26.93	32.60	ND	20.44	31.29	ND
miR-26a	21.93	27.93	ND	20.14	29.60	33.95
miR-140	30.29	31.37	36.53*	25.44	34.19	ND
miR-212	21.66	20.30	31.51	29.28	37.11	32.75
miR-423-3p	19.87	25.63	32.28*	ND	ND	ND
miR-708	24.75	28.28	37.43*	21.87	30.84	ND
miR-767	16.16	21.72	34.88*	ND	ND	ND

Numbers represent Ct values. ND = not detected , * Detected in URT, but not detected in the TaqMan platform.

### Subject recruitment

A series of 351 consenting individuals destined for lung sampling for clinical purposes (bronchoscopy or thoracic surgery) were enrolled under a protocol approved by the Einstein-Montefiore institutional review board (IRB). All subjects provided informed consent for participation and publication. All study methods and protocols were carried out in accordance with the institutional Einstein-Montefiore IRB guidelines and regulations. This observational series work was PRoBE compliant^37^. This study included 166 cases of lung cancer and 185 controls without lung cancer (Table 2). It also included 4 healthy volunteers from whom EBC was collected at three different timepoints (0, 24, and 96 h). EBC (and other non-invasive airway specimen) collection occurred immediately prior to the planned bronchoscopy/thoracic surgery, to preclude procedure-induced spillage of lung materials into the EBC (and mouthwash) samples. Clinical data was obtained by direct interview, also in advance of any clinically-indicated bronchoscopic/surgical procedure (and therefore in advance of tissue diagnosis), and verified manually in the clinical electronic medical record. Inclusions were: age > 21; fitness for the clinically-indicated (bronchoscopy/surgical) procedure; capacity and willingness to consent. Exclusions were: acute respiratory illness, contraindications to additional brushings/bronchoalveolar lavage (coagulopathy/known poorly controlled uremia); lack of capacity for consent. As such, subjects entailed a diversity of ages, ethnicities, smoking histories, clinical diagnoses, and underlying chronic lung diseases, which were accounted for in the models.

**Table 2:** Clinical characteristics among 351 cases and controls.

	Cases (n = 166)	Controls (n = 185)	Statistic	*p*-value
Age (years)	66.93	56.40	t-test	1.23E−15
Gender (% male)	48.80	49.73	chi-square	0.86
Smoking Status (%)	overall		chi-square	1.51E−10
Current	43.37	20.54		
Former	47.59	41.62		
Never	9.04	37.84		
Pack Years	43.43	19.21	t-test	3.12E−09
Quit Years (former smokers)	7.33	9.40	t-test	6.00E−03
Pack years-Quit Years	31.14	5.82	t-test	7.32E−06
Tumor Histology (%)			N/A	
Adeno	50.0	N/A		
Squam	21.1	N/A		
Undiff NSCLC	15.7	N/A		
Small Cell	9.0	N/A		
Mets/Other	4.2	N/A		
Stage (%)			N/A	
I	33.13	N/A		
II	12.05	N/A		
III	31.33	N/A		
IV	11.45	N/A		
ULD (%)				
COPD	56.02	19.46	Fisher	1.29E−12
Fibrosis	1.20	2.16	Fisher	0.69
Inflammation, NOS	1.20	10.27	Fisher	2.16E−04
Asthma	12.05	16.76	Fisher	0.23
Sarcoid	1.20	8.65	Fisher	1.30E−03
Bronchiectasis	2.41	2.70	Fisher	1.00
None	31.33	49.19	Fisher	7.00E−04

Clinical characteristics of the case versus control subjects. Former smoker, defined as quit > 1 year; COPD, defined clinically (MD report, medications), radiographically, and/or pathologically (where available) in medical records; Pack-yrs – Quit-yrs, in former smokers, a constructed composite tobacco exposure risk variable combining cumulative dose (pack years) minus proximity/recency of smoking (quit-years); NOS, not otherwise specified. Adeno = adenocarcinoma; Squamous = squamous cell carcinoma; NSCLC-Undifferentiated non-small cell lung cancer; Small cell = small cell carcinoma; Mets/Other = metastases from other organs to lung or other tumor histologies. ULD = Underlying (chronic) lung disease.

### Statistical analysis

Logistic Regression (LR): Logistic Regression was performed for each miRNA with cancer case–control status as the response, with and without clinical variables included as the covariates. The clinical covariates were age, gender, smoking status (never smokers, former smokers, current smokers), pack years, quit years, and underlying lung diseases. To reduce dimensionality, underlying diseases were categorized into three groups, based on the distribution of these disorders in our cohort:(1) known carcinogenesis risk: any of COPD, fibrosis, generic inflammation and/or asthma; (2) sarcoid and bronchiectasis; (3) none and others.

Random Forests (RF): Two types of Random Forest^38,39^ classifiers were built for comparison, using R package random forest^39^. First, an RF classifier was built on the clinical variables alone: age, gender, smoking status, pack-years, quit-years, underlying lung disease (type), tumor histology, stage. Two-fold cross-validation was repeated 20 times to gauge the accuracy of this classifier, and its sensitivity, specificity, positive and negative predictive value. Second, an RF classifier was built on the clinical variable plus the microRNA variables together. To compare the performance of the two types of RF classifiers, we further generated 100 resampled ROC curves for each classifier and compared the average area under the curve (AUC) between the two models using a two-independent sample t-test. A resampled ROC curve was generated by repeatedly splitting the dataset into 50% training, 50% testing (100 times), building the two random forest models (clinical and clinical + microRNA), and predicting the outcomes of the testing split.

Airway topography similarity statistic: A subset of 12 EBC donors provided bronchoscopic samplesets of deep alveolar (BAL) and major airway (bronchial, BB) levels, as well as sputum and mouthrinse. The pilot sub-study (Supplemental Table S3) was designed to evaluate if an individual microRNA profile from EBC retains the distinct features of the microRNA profile from deep lung (bronchial brushings or bronchoalveolar lavage), or alternately resembles contaminating upper airway/mouthwash tissues. This was done by applying an arbitrary panel of 13-microRNAs interrogated by qualitative RT-PCR against samples from 12 individuals, each donating five airway level samples for comparison [bronchoalveolar lavage (BAL), bronchial brush (BB), sputum (SP), mouthrinse (MW), EBC]. To statistically test the surrogacy of EBC-microRNA for deeper lung specimens (bronchial brushings and bronchoalveolar lavage), we developed a similarity statistic of two tissue types based on Hamming distance. That is $\mathrm{SH}= \sum_{\mathrm{i}}\mathrm{H}\left({\mathrm{d}}_{\mathrm{i}}, {\mathrm{d}}_{\mathrm{i}}^{\mathrm{^{\prime}}}\right)$, where ${\mathrm{d}}_{\mathrm{i}}$ and ${\mathrm{d}}_{\mathrm{i}}^{\mathrm{^{\prime}}}$ are (binary) miRNA profiles from two tissue types of the same individual i. The Hamming distance H gives the total number of miRNAs for which the two profiles d and d’ are discordant. The smaller the statistic SH is, the more similar are two tissue types in miRNA profiles within each subject. If the two tissue types from the same individual are not closer than two tissue types from two random individuals, then there is no information in one of the tissues to infer the miRNA profile of the other tissue. To test that the two tissues from the same individuals are closer than two random individuals, we performed a permutation test that permutes the miRNA profiles within each tissue type among individuals.

Temporal stability of EBC miRNA for an individual across time: The EBC samples from 4 healthy volunteers at three different timepoints were used to evaluate temporal stability of EBC miRNA for an individual across time. The three targets were miR-141, miR-142-3p and miR-205 and the internal housekeeping gene was miR-423-3p. Heat matrix was built by delta Ct (normalizing Ct of target miRNA to Ct of housekeeping miR-423-3p). The total qPCR cycles was 40. For the housekeeping miR-423-3p, the Ct cutoff was 35 and the melting curve of SYBR green qPCR must be correct.

## Results

Optimization results from miRNA extraction to reverse transcription to PCR are described below.

### microRNA extraction and ethanol precipitation

In Supplemental Figure S1A, different extraction platforms are tested. Graphs depict quantitative realtime RT-PCR melt curve with SYBR green intercalating dye. Ethanol precipitation (followed by Trizol) appeared to be optimal means to extract miRNA from EBC, judging by lower Ct value – the lower the Ct value, the earlier the amplified PCR product signal reaches a given threshold fluorescence, indicating higher starting template.

#### Carriers

In Supplemental Figure S1B, microRNA isolation carrier molecules were tested, glycogen versus RNA. Quantitative RT-PCR melt curve with SYBR green intercalating dye. Ethanol precipitation with either glycogen or RNA carrier performed similarly in extracting miRNA from EBC, judging by similar Ct values.

### Specificity testing of any newly designed microRNA primer

All microRNA primer designs passed the four specificity tests, viewed by gel electropheresis of final PCR product and key controls (Fig. 1B)). These tests included: (a) no-RT step (r/o DNA-derived signal); (b) no polyA-step (r/o messenger RNA -derived signal); (c) genomic DNA spike in (r/o gDNA derived signal); (d) water-no template blank (r/o cDNA or PCR-product contaminated reagents).

### Universal RT primer optimization for microRNAs

It was demonstrated (Supplemental Figure S2) that different URT designs confer different sensitivity (by CT differences, final PCR products), and different discrimination of microRNA versus mRNA (by CT differences, final PCR products between the microRNA (polyadenylated) and mRNA (no polyadenylation) conditions.

### Further distinguishing miRNA from mRNA based on different URTs

Supplemental Table S1 depicts microRNA specificity, expressed as delta CT of messenger RNA (no polyadenylation step included) versus microRNA product (polyadenylation step included) by realtime PCR. The greater the difference (deltaCT) between the two conditions, the more microRNA-specific is the microRNA primerset. Melt curve analysis was also analysed (Supplemental Figure S2). By these criteria, UPRT*_V and UPRT-4 are both adequate URTs, UPRT*_V was slightly advantaged over UPRT-4 based on 24 miRNA primer sets, overall. Acceptable microRNA versus mRNA discrimination listing delta-CT between these two co-amplified species is tabulated in this figure.

### URT-PCR sensitivity and specificity

**Figure 2:**
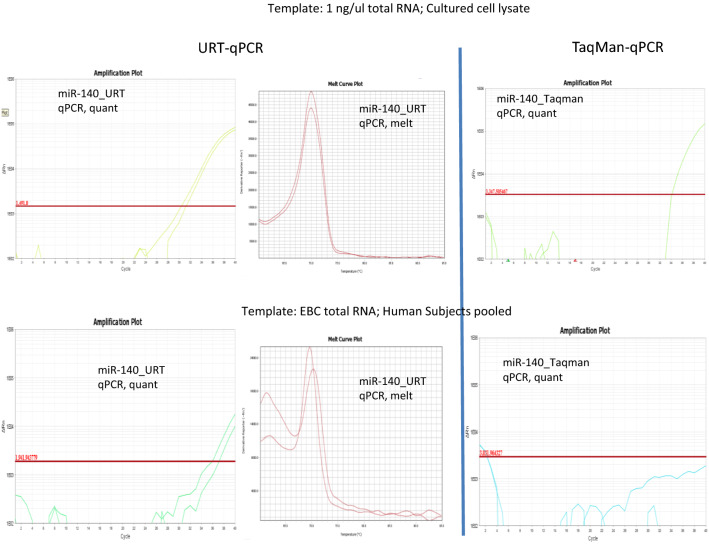
Verification of URT-PCR sensitivity. The relative sensitivity of the URT-PCR strategy was tested against the commercial standard (probe-based qRT-PCR (TaqMan). For six of ten direct comparisons, URT-PCR detected trqnscript, but TaqMan did not; examples (Panel **A**) miR-140 and (Panel **B**) miR-423-3p are shown. *(Top)* Total RNA (1 ng/ul) from a mixture of epithelial lung cells pooled was interrogated by both methods; URT-qPCR (left) showed CT 27 versus 31 for TaqMan, or 2^4 = 16-fold more sensitive. *(Bottom)* Total RNA extracted from EBC by conventional means (Methods) showed miR 423-3p as detectable by URT-qPCR, but absent by TaqMan).

The specificity and sensitivity were compared between URT-qPCR and Taqman qPCR. Positive controls and negative controls, including cDNA of mRNA and miRNA, minus RT, cDNA of mRNA, genomic DNA and water were used to identify an optimal primer pair with microRNA specificity in URT-qPCR. As for specificity of qPCR, both URT-qPCR and Taqman qPCR can be specific for targets (Fig. 2). As for sensitivity of qPCR, URT-qPCR was more sensitive than Taqman qPCR for some low copies templates (let-7a, miR-18a, miR-140, miR-423-3p, miR-708, miR-767 (Table 1).

### EBC surrogacy for the lung

The similarity statistic of two tissue types (Supplemental Table S3) were based on Hamming distance, $\mathrm{SH}= \sum_{\mathrm{i}}\mathrm{H}\left({\mathrm{d}}_{\mathrm{i}}, {\mathrm{d}}_{\mathrm{i}}^{\mathrm{^{\prime}}}\right)$, and 1000 permutations of miRNA profiles within each tissue type among individuals, gave an estimated *P*-value of 0.007, suggesting that the miRNA profiles of EBC are closer to miRNA profiles of BAL of the same individual than to miRNA profiles of BAL of random individuals. The same analysis was applied between EBC and BB (*p* = 0.23), EBC and SP (*p* = 0.18), EBC and MW (*p* = 0.04).

### The clinical characteristics of the 351 subjects

The clinical characteristics of the 351 subjects are described in Table 2. Baseline clinical characteristics that differed between cases and controls included age, smoking status, pack-years, quit years, underlying lung disease. Former smokers were defined as self-reported quit greater than one year from enrollment. Cases significantly differed from controls for: age (66.9 vs. 56.4, resp.); smoking status (current 43.4 vs. 20.5% , former 47.6% vs. 41.6%, never 9.0 vs. 37.8% never smokers); pack-years among current/former smokers (43.4 vs. 19.2); quit years among former smokers (7.3 vs. 9.4); pack years-quit years index (31.1 vs. 5.8); underlying lung disease including COPD % (56.0 vs. 19.5); inflammation NOS% (1.2 vs. 10.3); sarcoidosis% (1.2 vs. 8.6); none% (31.8 vs. 49.2). Both logistic regression (LR) and random forest (RF) discriminant models took these clinical inter-group differences into account. For RF, this included measuring the incremental impact on case–control discrimination of microRNAs over and above these clinical factors alone.

### Logistic regression

LR models were created (Table 3), using individual exhaled microRNA presence or absence as univariate predictors of case–control status, with adjustment for clinical factors: age, gender, smoking status (current, former, never), smoking pack years and quit years, and presence of underlying lung disease. For the entire data set, miR-21, 33b and 212 appeared to be somewhat informative for case–control status (*p* < 0.05), after adjustment for the above-listed clinical factors.

**Table 3:** Logistic regression, univariate miR, All subjects, n = 351.

##	miRNA	*p*	p.adj
1	miR.324.5p	0.967	0.817
2	miR.9	0.383	0.779
3	miR.21	0.090	0.020
4	miR.31	0.343	0.231
5	miR.33b	0.011	0.017
6	miR.96	0.631	0.342
7	miR.105	0.677	0.142
8	miR.146a.5p	0.358	0.340
9	miR.182.5p	0.840	0.687
10	miR.196b	0.385	0.587
11	miR.199b.5p	0.640	0.562
12	miR.200a	0.799	0.396
13	miR.200b	1.000	0.959
14	miR.205	0.664	0.793
15	miR.212	0.153	0.033
16	miR.221	0.932	0.386
17	miR.345	0.113	0.081
18	miR.429	1.000	0.601
19	miR.767	0.236	0.476
20	miR.944	0.053	0.404
21	miR.1269a	0.059	0.496
22	miR.1293	0.102	0.230
23	miR.1910	0.261	0.154
24	miR.3662	0.862	0.539

The univariate models included adjustments for clinical factors of age, gender, smoking status (current, former, never), smoking pack-years and quit-years, and underlying lung disease. Underlying lung disease: For all models, underlying lung disease was treated as trichotomous (COPD /fibrosis//inflammation NOS, asthma) *versus* sarcoidosis/bronchiectasis) *versus* none/other. Housekeeper transcript miR423-5p is not listed.

### Random forests

**Figure 3:**
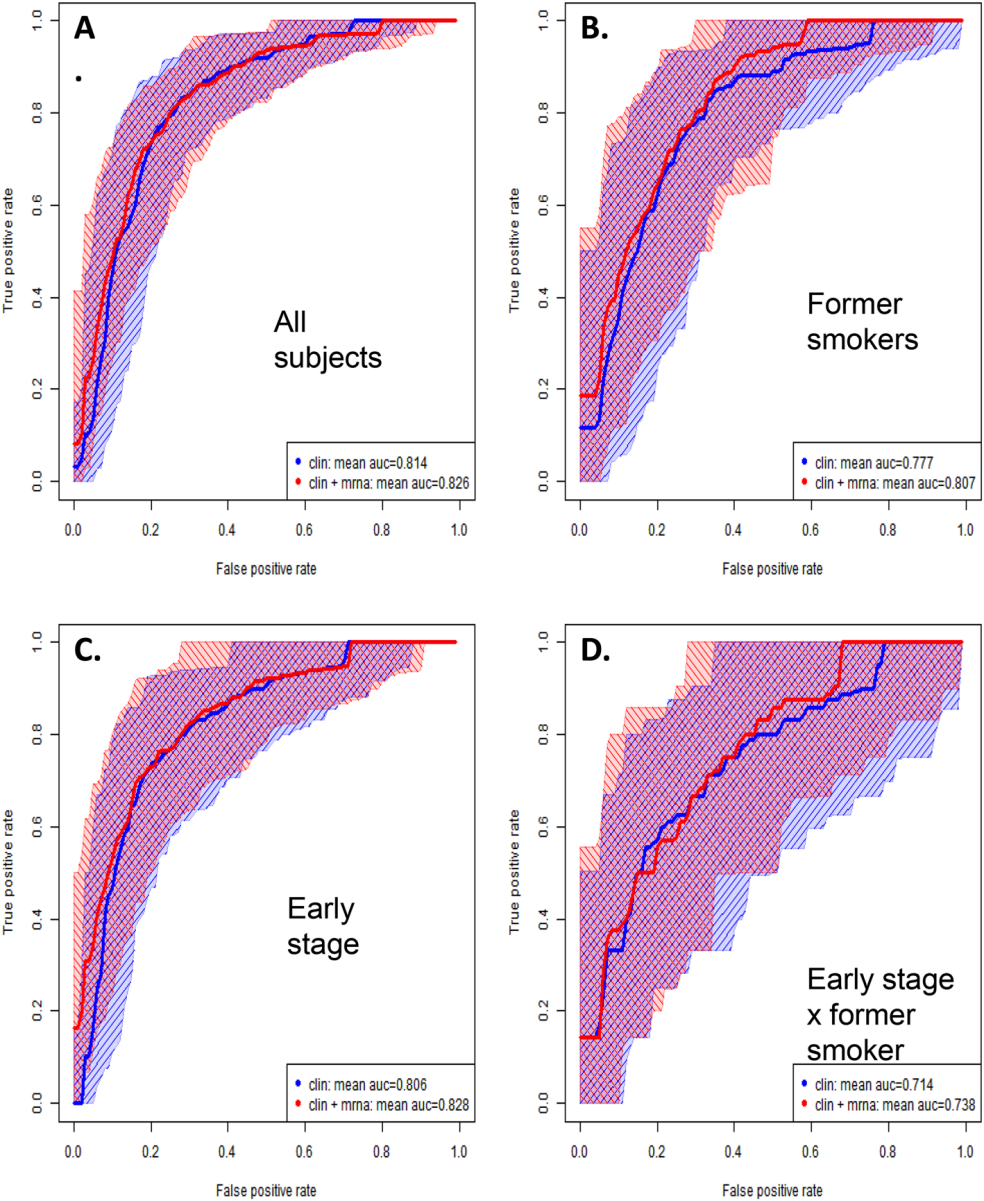
Receiver Operating Curves (ROC). Compare clinical factors alone model *versus* clinical factors + exhaled microRNAs combined model (red), to distinguish early stage (I + II) lung cancer all tumor histologies case donors combined, versus non-cancer controls. (**A**) All smoking categories combined; (**B**), Former smokers only; (**C**) Early stage only; (**D**) Early stage x former smokers. Random forests, recursive partitioning and cross-validation were employed as described in the statistical analysis section. This ROC plot of true positives versus false positives shows borderline incremental information (Fig. 3A, Clinical AUC + 1.2% (*p* = 0.07)) value of the exhaled microRNAs over and above the clinical model alone, in all subjects combining all smoking status’, stages, and histologies. It particularly held true in main subgroup analyses, separating out former smokers (Fig. 3B, Clinical AUC + 3.0% (*p* = 6.0e−03)), and in early stage (I,II) models (Fig. 3C, Clinical AUC + 2.2% (5.1e−03)). In combining these subgroups (**D**), early stage x former smokers combined did not show significant case–control discrimination (Fig. 3D, NS). Model components and significance testing of area under curve (AUC) differences are described in Table 4.

Clinical, exhaled microRNA, and combined clinical + exhaled microRNA RF models discriminating cases from controls were constructed (Table 4). For lung cancer overall, including all subjects (n = 351) and all case primary lung malignant tumor histologies, the clinical RF model included age, gender smoking status, pack-years, quit-years, underlying lung disease. For the *clinical only* RF model alone, case–control discriminant accuracy, sensitivity, specificity, positive predictive value, negative predictive value, AUC-ROC, were: 0.74, 0.74, 0.74, 0.76, 0.72, 0.814, respectively (Fig. 3A). For the *microRNAs only* model, the respective values were: 0.57, 0.63, 0.50, 0.58. 0.55, 0.611. For the *combined clinical* + *microRNA* model, the respective performance values were: 0.74, 0.74, 0.74, 0.76, 0.73, 0.826. The added AUC discrimination conferred by exhaled microRNAs for the overall group of subjects (n = 351) was 1.2% (0.814 =  > 0.826; *p* = 0.07, Welch t-test).

**Table 4:** Exhaled microRNA RF models.

RF models, lower stringency	Individual component factors	Accuracy (*p*-value)	Sensi, Speci	PPV, NPV	ROC-AUC	AUC difference, Clinical versus Clinical + micro-RNA, % (*p*-value)
All subjects, all smoking categories, all tumor histologies, n = 166 cases, 185 controls						
Clinical variables alone (unselected)	All clinical variables [age, gender, smoking status, pack-years, quit-years, underlying lung disease, tumor histology for cases]	0.74 (< 2.2e−16)	0.74, 0.74	0.76, 0.72	0.814	
microRNAs alone	All 24 microRNAs Important miRs: 21, 33b, 944, 1269a, 1910	0.57 (< 2.2e−16)	0.63, 0.50	0.58, 0.55	0.611	
Clinical + microRNA	All Clinical factors and All 24 miRs	0.74 (2e−16)	0.74, 0.74	0.76, 0.73	0.826	1.2% (0.07)
Former smokers only n = 79cases, 77 controls						
Clinical variables alone	All clinical variables [age, gender, smoking status, pack-years, quit-years, underlying lung disease]	0.69 (< 2.2e−16)	0.69 0.69	0.69 0.70	0.777	
microRNAs alone	All 24 microRNAs Important miRs: 33b, 146a.5p, 200a, 212, 1293	0.59 (< 2.0e−16)	0.57 0.61	0.59 0.59	0.656	
Clinical + microRNA	All Clinical and All miRs	0.70 (< 2.0e−16)	0.67 0.72	0.70 0.69	0.807	3.0% (6.0e−03)
Early stage only (stages I and II) n = 78 cases, 184 controls						
Clinical Variables Alone	All clinical variables [age, gender, smoking status, pack-years, quit-years, underlying lung disease]	0.75 (2.2e−16)	0.86 0.50	0.80, 0.60	0.806	
microRNAs alone	All 24 microRNAs Important miRs: 96, 146a.5p, 944, 1269a, 1910	0.700 (NS)	0.90, 0.23	0.73 0.49		
Clinical + microRNA	All Clinical variables and All miRs	0.76 (< 2.2e−16)	0.90, 0.45	0.79 0.65	0.828	(2.2% (5.1e−03)
Former Smoker x Early Stage Sub-subgroup n = 34 cases, 77 controls						
Clinical variables alone	All clinical variables [age, gender, smoking status, pack-years, quit-years, underlying lung disease, tumor histology for cases]	0.71 (1.9e−03)	0.87 0.35	0.75 0.54	0.714	
microRNAs alone	All 24 microRNAs Important miRs: 96, 146a.5p, 200b, 345, 1910	0.67 (NS)	0.86 0.25	0.72 0.44	0.641	
Clinical + microRNA	All Clinical and All miRs	0.71 (1.4e−02)	0.90 0.28	0.74 0.54	0.738	2.4% (NS)
Current Smokers only, n = 38cases, 72 controls						
Clinical variables alone	All clinical variables [age, gender, smoking status, pack-years, quit-years, underlying lung disease, tumor histology for cases]	0.78 (2.2e−16)	0.59 0.87	0.71 0.80	0.731	
microRNAs alone	All 24 microRNAs Important miRs: 105, 146a.5p, 182.5p, 200a, 205	0.59 (NS)	0.15 0.82	0.30 0.65	0.464	
Clinical + microRNA	All Clinical and All miRs	0.76 (2.2e−16)	0.48 0.90	0.73 0.77	0.764	3.3% (3.5e−02)
Adenocarcinoma only, n = 87cases, 184 controls						
Clinical variables alone	All clinical variables [age, gender, smoking status, pack-years, quit-years, underlying lung disease]	0.74 (< 2.2e−16)	0.83 0.53	0.79 0.60	0.817	
microRNAs alone	All 24 microRNAs Important miRs: 96, 146a.5p, 221, 944, 1269a	0.65 (NS)	0.87 0.18	0.69 0.40	0.579	
Clinical + microRNA	All Clinical and All miRs	0.73 (2.2e−16)	0.87 0.43	0.76 0.61	0.796	−2.1% (1.1e−02), neg direction
Late Stage (III, IV) only, n = 77cases, 184 controls						
Clinical variables alone	All clinical variables [age, gender, smoking status, pack-years, quit years, underlying lung disease, underlying lung disease]	0.74 (2.2e−16)	0.84 0.49	0.80 0.56	0.797	
microRNAs alone	All 24 microRNAs Important miRs: 31, 33b, 105, 212, 944	0.67 (NS)	0.90 0.12	0.71 0.35	0.541	
Clinical + microRNA	All Clinical and All miRs	0.74 (2.2e−16)	089 0.38	0.77 0.58	0.809	1.2% (NS)
Late Stage (III, IV) x Former Smoker, n = 40 cases, 77 controls						
Clinical variables alone	All clinical variables [age, gender, smoking status, pack-years, quit years, underlying lung disease, underlying lung disease]* histologies?]*	0.70 (1.6e−13)	0.82 0.46	0.75 0.57	0.781	
microRNAs alone	All 24 microRNAs Important miRs: 33b, 200a, 212, 345, 1293	0.63 (NS)	0.84 0.22	0.67 0.41	0.654	
Clinical + microRNA	All Clinical and All miRs	0.69 (1.1e−07)	0.85 0.37	0.72 0.57	0.789	0.8% (NS)

RF Models of case–control distinction, including clinical variables alone, exhaled microRNAs alone, and the two combined, and performance characteristics of accuracy, sensitivity, specificity, positive and negative predictive value (PPV, NPV, resp.), Underlying lung disease is treated as a (trichotomous) variable: (3). COPD or fibrosis or inflammation or asthma (all carry some lung cancer risk); versus (2). sarcoid bronchiectasis; versus (1) none/other).

For a priori selected subgroups, data analyses are also tabulated (Table 4). For example, *Former smoker* cases versus former smoker controls comparison of case–control discriminant performance were again described in terms of discriminant accuracy, sensitivity, specificity, positive predictive value, negative predictive value, AUC-ROC (Fig. 3B). For the *clinical only* model, performance parameters were: 0.69, 0.69, 0.69, 0.69, 0.70, 0.777, respectively. For the *microRNA-only* model, performance parameters were: 0.59, 0.57, 0.61, 0.59, 0.59, 0.656, respectively. For the *combined clinical* + *microRNA* model, performance parameters were: 0.70, 0.67, 0.72, 0.70, 0.69, 0.807, respectively. The added AUC discrimination conferred by exhaled microRNAs was 3.0% (0.777 =  > 0.807; *p* = 6.0e−03, Welch t-test) for former smokers. Similarly, early stage (I + II combined) cases showed 2.2% added case–control AUC discrimination from the exhaled microRNA panel (*p* = 5.1e−03) (Fig. 3C). For additional clinically important combined subgroups, the case versus controls models’ performance characteristics are described in Table 4, Fig. 3A–D and Supplemental Fig. 3A–D.

### Temporal stability of EBC miRNA for an individual across time

Three target miRNAs (miR-141, miR-142-3p and miR-205) in EBC at three timepoints (0, 24, 96 h) of four individuals were able to be detected by realtime PCR and normalized to housekeeping miR-423-3p. Data suggests that the EBC samples at different timepoints in the same subject were stable to a large extent (Supplemental Figure S4).

## Discussion

This report describes initial steps in exhaled microRNA platform establishment and optimization. In application, it represents the most comprehensive interrogation of microRNAs in exhaled breath to date, here performed to distinguish subjects with and without primary lung cancers^17^. Starting with a lung tissue-based microRNA-seq discovery effort combined with published literature-suggested microRNAs, we interrogated a panel of 25 microRNAs in exhaled breath condensate using our RNA-specific qualitative RT-PCR. We found that: (i) microRNAs are detectable in exhaled breath condensate; (ii) there are individual exhaled microRNAs that offer some case–control discrimination by logistic regression (microRNAs 21, 33b, 212), and (iii) additional RF models were developed, using the entire microRNA panel, that also suggest some modest additional case–control discrimination, particularly in the subsets of former smoker, and early stage subjects, over and above that demonstrated in comprehensive clinical models.

Technical challenges abound in examining nucleic acids in exhaled breath. While EBC is widely available non-invasively, this breath specimen entails only trace levels of suspended microRNA template, at the sub-picomole/femtomole level. This is perhaps because the nucleic acid templates are by definition, higher in molecular weight (20–22 nucleotides in length, > 200 carbons) than is typically true for smaller exhaled condensate-suspended (e.g., small polar metabolites), or labile VOC gas-phase molecules (e.g. H_2_O_2_, 8-isoprostane, others) . Nonetheless, the PCR confers capacity for detection of microRNAs at this low template copy level, as is suggested here. The trace concentrations inherent to EBC specimens for most analytes, including nucleic acids has, to-date, precluded performing discovery efforts such as microRNA next generation sequencing, and to date reliable quantitation, directly from this matrix, although methods are evolving.

The microRNA interrogation panel choice was therefore based on: (i) a previously unpublished microRNA seq effort (ENA accession: PRJEB52036) inter-tissue comparison of 32 lung resected bronchogenic carcinoma versus remote lung tissue (stratified for adenocarcinoma, squamous cell carcinoma histologies), with 10 representative overexpressed microRNAs included from each of those two most common histologies. The remainder came from: (ii) TCGA^15,16^; and (iii) several literature-identified microRNA markers of lung cancer. This lung tissue-based interrogation served as a plausible starting point for assembly of an initial candidate list of potentially exhaled breath donor discriminant microRNAs, in the absence of technical capacity for discovery in EBC itself. We used our previously published microRNA-PCR that is micro/mRNA–specific, as it excludes gDNA fragment false priming by employing a uniquely tagged RT-primer strategy^21–23^ and in primer design precluded false amplification of messenger RNA fragments. It appeared more sensitive than a commonly used commercial probe based microRNA PCR platform (TaqMan, Invitrogen). We chose to treat the data as qualitative (individual miR, present/absent) because we were insufficiently confident of robust quantitative RT-PCR data in the absence of a robustly quantifiable internal housekeeper at these trace levels. Performance of the fluorescent intercalating (SYBR) dye detection strategy coupled to URT-PCR on the realtime PCR platform did allow quality assurance using the clarity of the fluorescence curve, melt-curve, and melt temperature with each PCR reaction. This was superimposed on a series of other analyses invoked during the design phase for primers, using multiple positive and negative controls, described in the Methods and Additional Studies sections.

This cross-sectional case–control design was chosen as it is accepted as a typical initial step in early development of potential risk biomarkers^40,41^. Clinical-demographic differences were observed in cases versus controls for age, smoking, pack-years, quit years, a pack-years minus quit-years composite index, underlying lung disease (COPD, inflammation/fibrosis, asthma, sarcoidosis, bronchiectasis). However, these clinical parameter case–control differences were equally modelled in both clinical-only models and in the clinical + microRNA combined models identically, so they should not have biased the incremental microRNA-attributable risk prediction. We emphasized enrollment of current and former smokers predominantly, as they are at markedly elevated risk for lung cancer, and therefore commonly come to clinical attention for surveillance, biopsy/resection. Our case and control ascertainment was crisp, minimizing misclassification as subjects were all confirmed histologically by virtue of their bronchoscopic/surgical procedures. Each subject underwent further verification of case and control status by an additional 3–6 month period of clinical follow-up, facilitated by electronically-retrieved clinical assessments from the engaged clinical pathologists, radiologists, surgeons, and pulmonologists on each subject. Recruited subjects with disputed case–control ascertainment (< 1% of enrolled) were excluded from the study.

In this moderate size case–control subject set, with an already selected candidate 25-microRNA panel (including housekeeper), we initially performed logistic regression, using case–control status as the main outcome variable, and a clinical model tested with/without each individual miR on the panel. Separately, we then employed iterative cross validation by random forests to assure stability of our results, rather than separate discovery and validation sets. The RF approach iteratively and randomly splits the data, substantively cross-validating in truly random fashion, and minimizing over-fit.

The clinical versus clinical-microRNA incremental differences are admittedly modest (~ 0.0–3.0%). We surmise that this is, in part, due to the strength of the clinical model alone displaying ROCs ~ 0.75–80, narrowing the clinical versus clinical + microRNA ROC performance gap. These were unusually robust clinical models, we believe for two reasons. First is the clinical model comprehensiveness, in part attributable to inclusion of all major known substantive risk factors for lung cancer (including quit years, underlying lung disease, others). Secondly, there is positive selection inherent to enrolling clinical bronchoscopy and surgical subjects such as these (above). That is, both (case and control) sets of subjects are drawn from the same base (procedurally-destined) population that is itself selected on clinical criteria to be at high risk for lung cancer. By definition, that high risk is perceived by the clinician as sufficient to warrant an invasive diagnostic/therapeutic procedure. Both of these factors (clinical model comprehensiveness, and clinical procedure-based enrollment selection) contribute to high risk in this clinical series, and imply that the clinical risk model performance would be elevated. Thus, the difference between this comprehensive clinical model alone, and that for this clinical model plus microRNA could potentially be artificially narrowed (as compared to that using conventional sparse clinical models of age and smoking status alone). We believe the negative impact of such selection on the estimate of the actual contribution of exhaled microRNAs to case–control discrimination, is counter-balanced by the rigor inherent in using the same (robust) clinical model when comparing clinical-only models versus combined clinical + microRNA models. Additionally, the definitive diagnoses inherent in recruiting those destined for lung sampling/pathologic readout was a strength. Overall, then, the above considerations suggest that ours is a conservative estimate of this initial, qualitative exhaled microRNA biomarker contribution in un-fractionated EBC, in real clinical conditions.

Among study limitations, we were compelled to use a dichotomous (present/absent) signal for a given microRNA in a given whole/unfractionated EBC sample, despite the reaction being run on a real-time PCR machine, for technical reasons. The real-time CT values, using the chosen platform for sensitivity but true of TaqMan as well, were not robust enough to generate reliable quantitative data. This issue is worth re-addressing in technical optimization studies, which are ongoing.

Additionally, the discriminant microRNA signal may in fact be small in magnitude, as our data suggest. This small magnitude of microRNA change in the “field” of cytologically normal bronchial epithelium itself has been suggested in a comprehensive RNA-seq study of bronchial brushings in a similar case control setting^14^. Notably, of the discriminant four bronchial epithelial case–control discriminant microRNAs in that report, only one (miR-146-5p) was interrogated in our study. While miR-146-5p was not individually case–control discriminant in our LR univariate models, it was contributory in the RF models for former smokers and early stage. There is a more recent smaller pilot report that EBC miRNAs might allow the identification, stratification and monitoring of lung cancer^38^.

We set out to survey the “state of the epithelium” i.e. the broad field of early carcinogenesis, rather than detection of a small peripheral cancer itself. This view of broad epithelial “field” interrogation is appropriate to risk assessment, rather than of a tumor detection/diagnostic tool. That the microRNA-based risk signal is likely from the broad field of histologically normal epithelium of the lung by sheer surface area considerations, rather than spillage from a small tumor, is also supported by the observation that early stage subset showed more case–control discrimination than the late stage cases, which would not be expected if the (larger) tumors were spilling (more) microRNA material.

## Conclusion

In conclusion, this is one of the first reports, and the most comprehensive report, of exhaled microRNAs in lung cancer case–control discrimination. Given the significant clinical potential, as well as the technical demands of this application, we plan to refine the exhaled microRNA interrogation technique, including sample processing and partition, miR-PCR quantitation, and microRNA panel adjustments, to better serve case–control discrimination. Assuming improved performance with these refinements, risk assessment efforts can be pursued in future prospective cohorts. Such trials could evaluate whether the biomarker platform can better predict future events, the “mother lode” of risk assessment^5^. If such utility was demonstrated, it would then allow for actionable clinical interventions, such as focusing early detection, or alternately perhaps directing chemoprevention, onto those individuals at highest risk.

## Supplementary Information

Supplementary Information.

## Data Availability

All data generated or analyzed during this study are included in this published article [and its supplementary information files]. The microRNA-seq datasets generated and/or analysed during the current study are available in the ENA repository [PRJEB52036].

## Abbreviations

TCGA: The cancer genome anatomy project. NCBI/NCI/NIH
AUC: Area under curve, for receiver operating curve (ROC)
ROC: Test performance plots sensitivity versus specificity
