# Supplementary material for: Initial development and testing of an exhaled microRNA detection strategy for lung cancer case–control discrimination
Source: Sci Rep. 2023 Apr 24;13:6620. doi: 10.1038/s41598-023-33698-8 (PMC10126132; doi:10.1038/s41598-023-33698-8)
Supplement: Supplementary file 1 — Supplementary Information. [file 41598_2023_33698_MOESM1_ESM.docx]

**SUPPLEMENTAL FIGURE LEGENDS:**

**
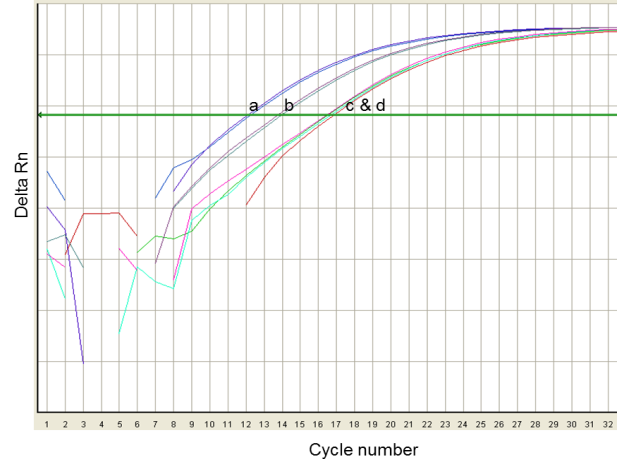

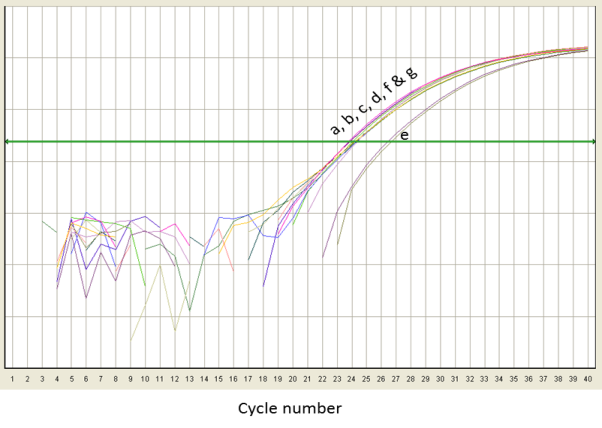
**

**A B**

**Supplemental Figure S1:**

**Figure S1A: Optimization of EBC microRNA extraction**. PCR product SYBR fluorescence is depicted on *y*-axis, cycle number on *x*-axis. Conditions: a. ethanol precipitated miRNAs; b. trizol-purified miRNAs; c. speed-vacuum concentrated miRNAs; d. column-purified miRNAs.

**Figure S1B: Optimization of nucleic acid ethanol precipitation conditions.** PCR product SYBR fluorescence is depicted on *y*-axis, cycle number on *x*-axis. Conditions: a. original miRNAs without ethanol precipitation; b. ethanol precipitated miRNAs with 20 ug glycogen.; c. ethanol precipitated miRNAs with 40 ug glycogen; d. ethanol precipitated miRNAs with 80 ug glycogen; e. ethanol precipitated miRNAs with 1 ug carrier RNA; f. ethanol precipitated miRNAs with 2 ug carrier RNA; g. ethanol precipitated miRNAs with 5 ug carrier RNA.

**A**

**
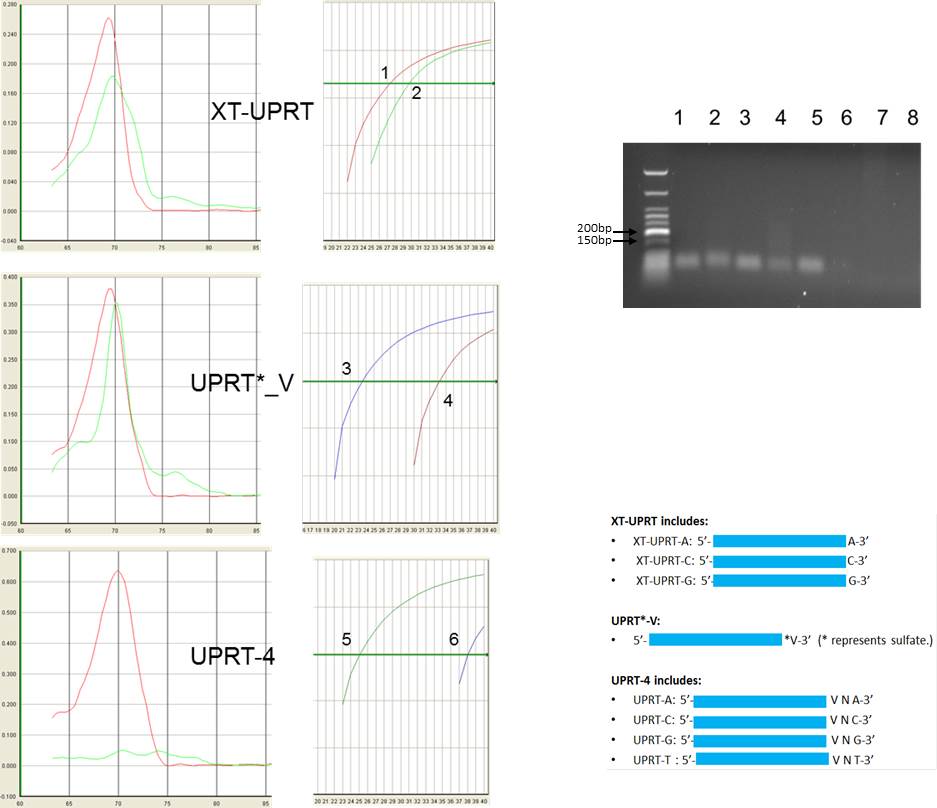
**

**C**

**B**

**Supplemental Figure S2:**

**Universal RT primer optimization for microRNAs specificity.** Among the URT options tested, XT-UPRT, UPRT*_V and UPRT-4 were potentially acceptable URT primers. (A) miRNA realtime PCR melting curve plot (left) and amplification plot (right). (B). agarose gel of different URT miRNA realtime PCR products. Lane 1: cDNA of microRNA (XT-URT); Lane 2: cDNA of mRNA (XT-URT); Lane 3: cDNA of microRNA (UPRT*-V); Lane 4: cDNA of mRNA (UPRT*-V) Lane 5: cDNA of microRNA (UPRT-4); Lane 6: cDNA of mRNA (UPRT-4); Lane 7: gDNA (A549 and Hela cell); Lane 8: water control. (C). Sequence differences among XT-UPRT, UPRT*-V and UPRT-4. In general, however, microRNA-size PCR products would not be expected to appear when PCR amplifying the cDNA of mRNA. However, such microRNA-size products were seen when amplifying the cDNA of mRNA produced by XT-UPRT and UPRT*_V, and the melting curves (A.top, middle) and PCR fragment sizes (B lanes 2,4) are very similar between cDNA of microRNA and cDNA of mRNA. For UPRT-4, there was no similar melting curve and no PCR band in cDNA of mRNA.


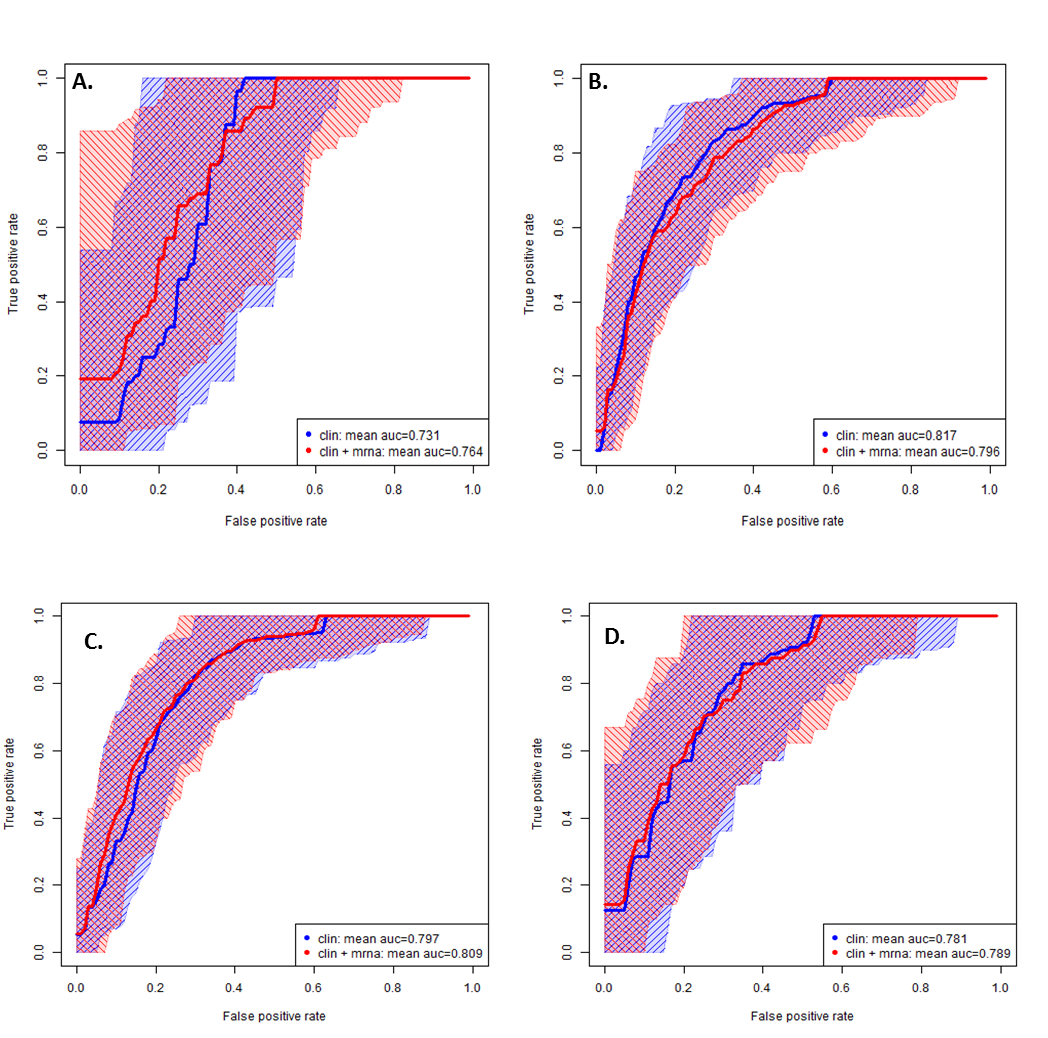


**Supplemental Figure S3**

**Subgroup analyses.** Random forests, recursive partitioning and cross-validation were employed as described in the statistical analysis section. For Current smokers (left, 3a), value of the exhaled microRNAs over and above the clinical model alone, was apparent Clinical model AUC+3.3% (p=3.5e-02)). For adenocarcinoma case subsets (right, 3b), the miR model detracted from the clinical+miR combined case-control discrimination, (Clinical AUC-2.1% (p= 1.1e-02). For all late stage cases (left lower, 3c), there was no significant additional case-control discrimination from exhaled microRNAs (Clinical AUC + 1.2% (NS)). For late stage cases who were former smokers (right lower 3d), there was no significant additional case-control discrimination from exhaled microRNAs (Clinical cAUC + 0.8% (NS)). Model component details and significance testing of area under curve (AUC) differences are described in **Main Table 4.**


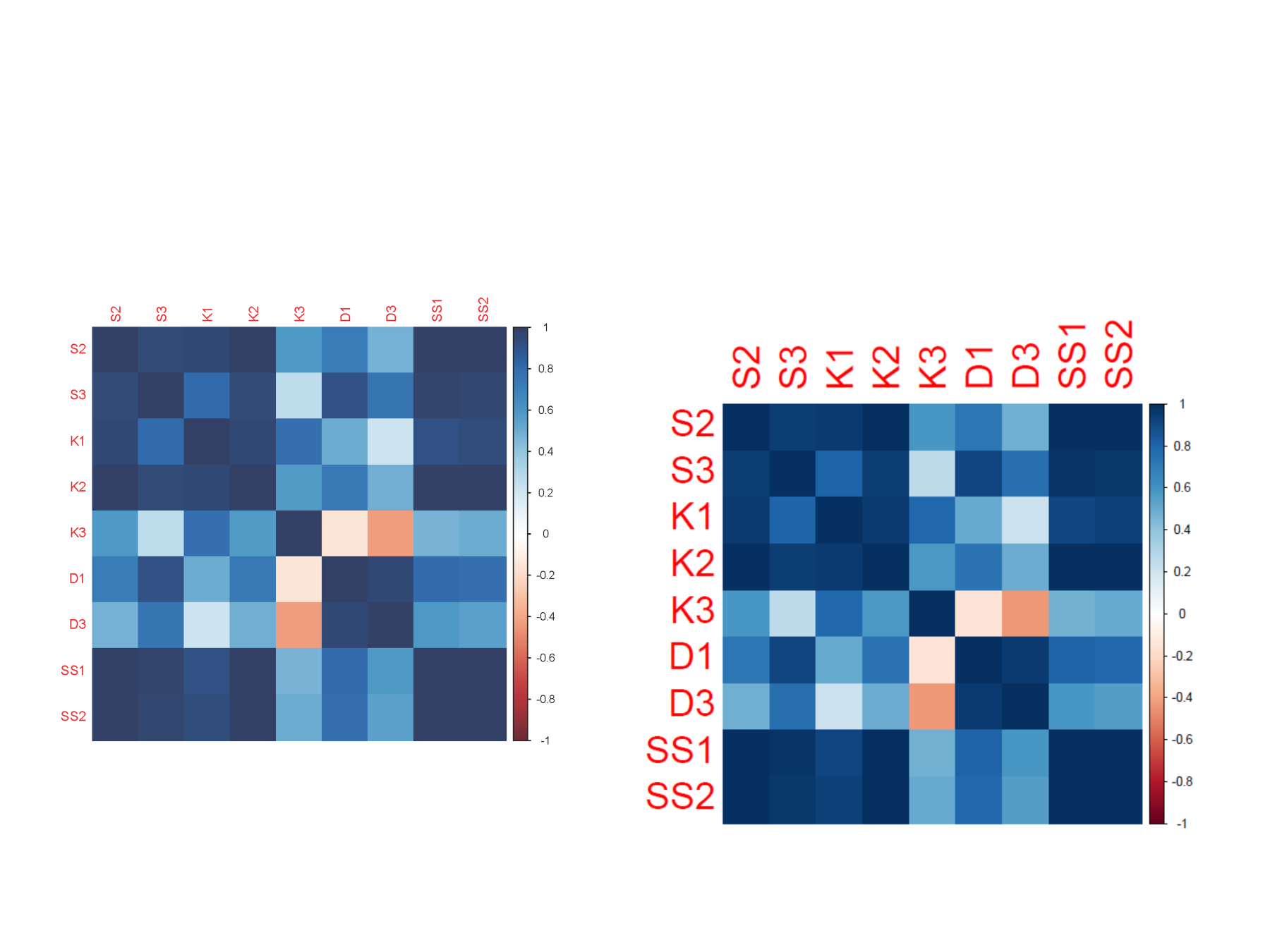


**Supplemental Figure S4**

**Temporal stability of EBC miRNA for an individual across time.** Heatmap depicts different (S, K, D and SS) volunteers, and S1, S2 and S3 represented different timepoints (0, 24, 96 hours) from each subject. Not all subjects were able to provide each timepoins. Correlation coefficients are ennumerated on right.

**
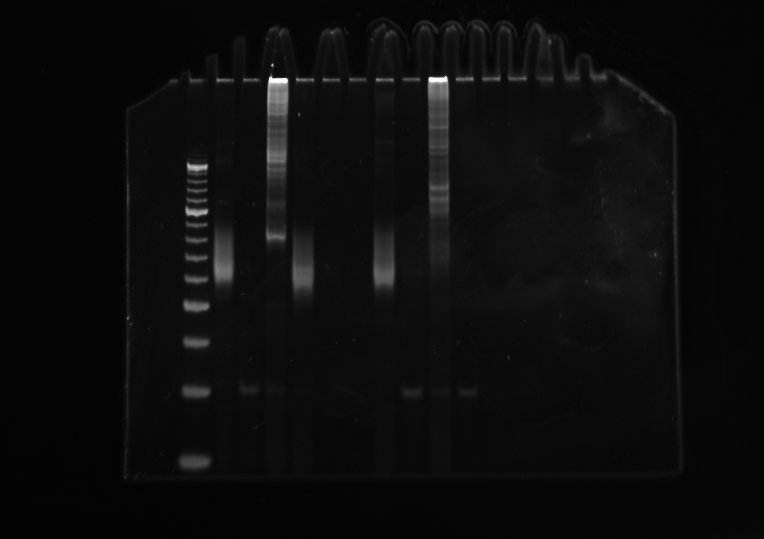
**

**Supplemental Figure S5**

**The original gel image.** The cut gel image in Figure 1B was from Supplemental Figure S5.


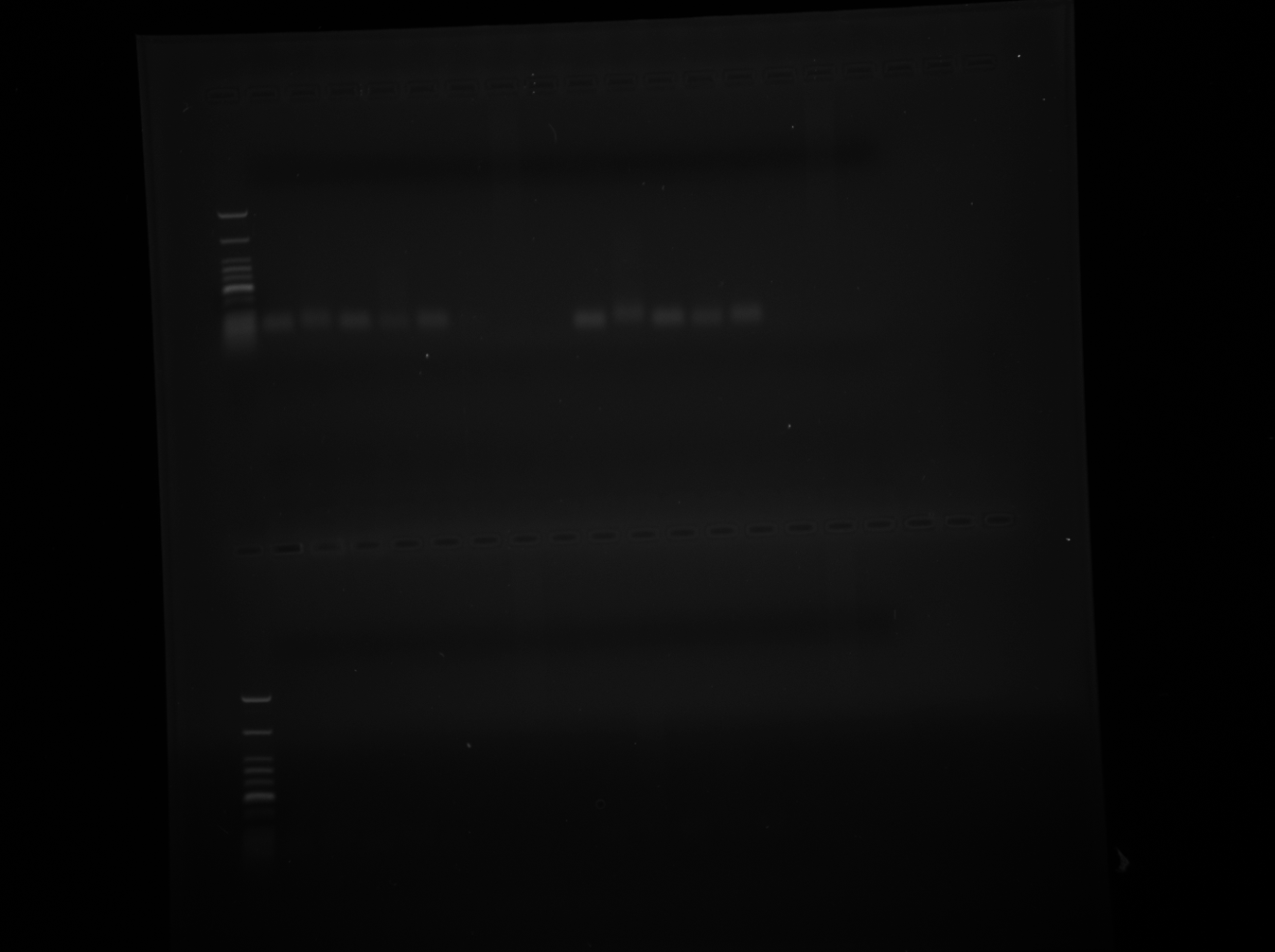


**Supplemental Figure S6**

**The original gel image.** The cut gel image in Supplemental Figure S2B was from Supplemental Figure S6.

**Supplemental Table S1. Distinguishing miRNA from mRNA based on different URTs.**

| miRNA forward primer | delta Ct(mRNA-miRNA) of XT-UPRT | delta Ct(mRNA-miRNA) of UPRT*_V | delta Ct(mRNA-miRNA) of UPRT-4 |
| --- | --- | --- | --- |
| 7_q1 | 3 | 10 | 14 |
| 9_N | 3 | 8.5 | 13.5 |
| 18a_q1 | 3.7 | 7.5 | 10 |
| 20a-5p_q2 | 1 | 9 | 9.5 |
| 21_q1 | 2.2 | 5.5 | 1.5 |
| 31_q1 | 5.7 | 12.4 | 14.1 |
| 126_q1 | 4.8 | 13 | 9.5 |
| 130b-3p_q1 | 0.5 | 6 | 5 |
| 135a_q2 | 1.5 | 8 | 3.5 |
| 142-5p_q1 | 4 | 1 | no signal |
| 146a-5p_q3 | 0.5 | 8 | 9 |
| 182-5p_q2 | 2 | 7.5 | 8 |
| 183-5p_q2 | 1.6 | 10 | 4 |
| 191_q1 | 1.2 | 12 | 8 |
| 196a-5p_q3 | 1 | 8.5 | 9 |
| 200a_q1 | 1.8 | 10.5 | 10.5 |
| 200c | 2.2 | 10.2 | 9.7 |
| 205 | 1.2 | 13 | 8 |
| 210_q3 | 3 | 4.3 | 4.3 |
| 212_q1 | 2.2 | 4.2 | 4.2 |
| 221 | 3.6 | 11 | 11.4 |
| 224_q3 | 2.6 | 12.7 | 13.8 |
| 330-3p_q3 | 4 | 1.5 | 2 |
| 708_q3 | 0.7 | 6.7 | 6.7 |

Supplemental Table S1 depicts microRNA specificity, expressed as delta CT of messenger RNA (no polyadenylation step included) versus microRNA product (polyadenylation step included) by realtime PCR. The greater the difference (deltaCT) between the two conditions, the more microRNA specific is the microRNA primerset.

**Supplemental Table S2. Primers, EBC-microRNA-PCR**

| primer name | sequence 5' - 3' | matched reverse primer |
| --- | --- | --- |
| miR-9 | CGATCTTTGGTTATCTAGCTGTAT | U60 |
| miR-21 | TAGCTTATCAGACTGAT | U60 |
| miR-31 | AGGCAAGATGCTGGCATAG | U60 |
| miR-33b | GTGCATTGCTGTTGCAT | U60 |
| miR-96 | TTTGGCACTAGCACATT | U60 |
| miR-105 | TCAAATGCTCAGACTCCTGTGG | U60 |
| miR-146a-5p | TGAGAACTGAATTCCATG | UR |
| miR-182-5p | TTTGGCAATGGTAGAACTCAC | U60 |
| miR-196b | TAGGTAGTTTCCTGTTGTTGG | U60 |
| miR-199b-5p | CCAGTGTTTAGACTATCTGTT | U60 |
| miR-200a | TAACACTGTCTGGTAACGATGT | U60 |
| miR-200b | TAATACTGCCTGGTAATGAT | U60 |
| miR-205 | CTTCATTCCACCGGAGTCT | U60 |
| miR-212 | TAACAGTCTCCAGTCACGGC | U60 |
| miR-221 | GCTACATTGTCTGCTGGGTT | U60 |
| miR-324-5p | GCATCCCCTAGGGCATTGG | U60 |
| miR-345 | GCTGACTCCTAGTCCAGGGC | U60 |
| miR-423-3p | CTCGGTCTGAGGCCCCTC | U60 |
| miR-429 | TAATACTGTCTGGTAAAACCG | U60 |
| miR-767 | TGCACCATGGTTGTCTGAGC | U60 |
| miR-944 | AAATTATTGTACATCGGATG | UR |
| miR-1269a | CTGGACTGAGCCGTGCTACTG | U60 |
| miR-1293 | TGGGTGGTCTGGAGATTTGT | U60 |
| miR-1910 | CCAGTCCTGTGCCTGCCGC | U60 |
| miR-3662 | GAAAATGATGAGTAGTGACTGAT | U60 |
| URT | AACGAGACGACGACAGACTTTTTTTTTTTTTTTTTTTTTV |  |
| U60 | AACGAGACGACGACAGACTTT |  |
| UR | AACGAGACGACGACAGAC |  |

Supplemental Table S2: Primersets used in the study.

**Supplemental Table S3. Airway level of origin patterns: EBC, MW, SP, BB, BAL; Summary table**

| **EBC**-Patient#  **(EBC)** | 7 | 9 | 18a | 21 | 31 | 126 | 191 | 200c | 205 | 210 | 212 | 221 | 224 | 708 |
| --- | --- | --- | --- | --- | --- | --- | --- | --- | --- | --- | --- | --- | --- | --- |
| **565** | - | + | - | - | - | - | - | - | - | + | + | + | - | - |
| **573** | - | - | - | - | - | - | - | + | - | + | + | - | + | - |
| **594** | + | - | - | - | - | - | - | - | - | - | + | - | - | - |
| **601** | + | - | + | - | - | - | - | - | - | - | + | + | - | - |
| **614** | + | - | - | - | - | - | - | - | - | + | + | - | - | + |
| **615** | - | + | + | - | - | - | - | - | - | + | + | - | - | - |
| **635** | - | - | - | - | + | + | - | + | + | + | + | + | + | - |
| **636** | - | - | + | - | - | - | - | + | + | + | + | + | + | + |
| **651** | - | + | + | - | + | - | + | - | + | - | + | - | + | - |
| **671** | - | - | - | - | + | - | - | + | - | + | - | + | - | - |
| **706** | + | - | - | - | + | - | + | + | + | + | + | + | - | + |
| **708** | - | - | - | - | + | - | - | - | + | + | - | + | - | - |
|  |  |  |  |  |  |  |  |  |  |  |  |  |  |  |
| **MW**-Patient#  **(MW)** | 7 | 9 | 18a | 21 | 31 | 126 | 191 | 200c | 205 | 210 | 212 | 221 | 224 | 708 |
| **565** | NA | NA | NA | NA | NA | NA | NA | NA | NA | NA | NA | NA | NA | NA |
| **573** | - | - | - | - | + | - | - | + | - | + | - | - | - | - |
| **594** | + | + | - | - | + | - | - | + | - | + | + | + | + | - |
| **601** | + | + | + | - | + | - | + | + | - | + | + | + | - | + |
| **614** | + | + | + | - | + | - | - | + | - | + | + | + | + | + |
| **615** | + | + | + | - | + | - | + | + | + | + | + | + | + | + |
| **635** | + | + | + | - | + | - | + | + | + | + | + | + | + | + |
| **636** | + | + | + | - | + | - | + | + | + | + | + | + | + | + |
| **651** | + | + | + | - | + | + | + | - | + | + | - | + | - | + |
| **671** | + | + | + | - | + | - | + | + | + | + | + | + | + | + |
| **706** | + | + | + | - | + | - | + | + | + | + | + | + | + | + |
| **708** | + | + | + | - | + | - | - | + | + | + | + | + | + | + |
|  |  |  |  |  |  |  |  |  |  |  |  |  |  |  |
| **SP**-Patient # **(SPUTUM)** | 7 | 9 | 18a | 21 | 31 | 126 | 191 | 200c | 205 | 210 | 212 | 221 | 224 | 708 |
| **565** | NA | NA | NA | NA | NA | NA | NA | NA | NA | NA | NA | NA | NA | NA |
| **573** | + | - | + | - | + | - | + | + | - | - | + | + | - | - |
| **594** | - | - | - | - | - | - | - | + | - | - | - | + | - | - |
| **610** | - | - | - | - | - | - | - | - | - | - | - | - | - | - |
| **614** | + | - | - | - | - | - | - | + | - | - | - | + | - | - |
| **615** | - | - | - | - | - | - | - | + | - | - | - | + | - | - |
| **635** | + | + | + | - | + | - | + | + | + | + | + | + | + | + |
| **636** | + | + | + | - | + | - | - | + | + | + | + | + | + | - |
| **651** | + | + | + | - | + | - | + | + | + | + | + | + | + | + |
| **671** | + | + | + | - | + | - | + | + | + | + | - | + | + | + |
| **706** | + | + | + | - | - | - | - | + | + | - | - | + | + | + |
| **708** | + | + | + | - | + | - | - | + | + | + | + | + | + | + |
|  |  |  |  |  |  |  |  |  |  |  |  |  |  |  |
| **BB**-Patient#  **(BB)** | 7 | 9 | 18a | 21 | 31 | 126 | 191 | 200c | 205 | 210 | 212 | 221 | 224 | 708 |
| **565** | - | - | - | - | - | - | - | - | - | - | - | - | - | - |
| **573** | + | + | + | + | + | + | + | + | - | + | - | + | + | + |
| **594** | + | + | + | + | + | - | + | + | + | + | + | + | + | + |
| **601** | + | + | + | + | + | + | + | + | + | + | + | + | + | + |
| **614** | - | - | - | - | - | - | - | - | - | - | - | - | - | - |
| **615** | + | + | + | - | + | + | + | + | - | + | - | + | + | + |
| **635** | + | + | + | + | + | + | + | + | + | + | + | + | + | + |
| **636** | + | + | + | + | + | + | + | + | + | + | + | + | + | + |
| **651** | + | + | + | + | + | + | + | + | + | + | + | + | + | + |
| **671** | + | + | + | + | + | + | + | + | + | + | + | + | + | + |
| **706** | + | + | + | + | + | + | + | + | + | + | + | + | + | + |
| **708** | + | + | + | + | + | + | + | + | + | + | + | + | + | + |
|  |  |  |  |  |  |  |  |  |  |  |  |  |  |  |
| **BAL**-Patient #  **(BAL)** | 7 | 9 | 18a | 21 | 31 | 126 | 191 | 200c | 205 | 210 | 212 | 221 | 224 | 708 |
| **565** | + | + | + | + | + | + | + | + | - | + | + | + | + | + |
| **573** | + | + | + | + | + | + | + | + | - | + | + | + | + | + |
| **594** | - | - | - | - | - | - | - | - | - | - | - | - | - | - |
| **601** | + | + | + | - | + | - | + | + | - | + | + | + | + | + |
| **614** | + | + | + | + | + | + | + | + | - | + | - | + | + | + |
| **615** | + | + | + | + | + | + | + | + | - | + | + | + | + | + |
| **635** | + | + | + | - | + | + | - | + | + | + | + | + | + | + |
| **636** | + | + | + | - | + | - | + | + | + | + | + | - | + | + |
| **651** | + | + | + | - | + | + | + | + | + | + | + | + | + | + |
| **671** | + | + | + | - | + | + | + | + | + | + | + | + | + | + |
| **706** | + | + | + | + | + | + | + | + | + | + | + | + | + | + |
| **708** | + | + | + | - | + | + | + | + | + | + | + | + | + | + |

Supplemental Table S3. EBC surrogacy for the lung: A subset of =12 EBC donors providing bronchoscopic samples of deep alveolar (BAL) and major airway (bronchial, BB) levels, as well as exhaled breath (EBC), mouthrinse (MW), sputum (SP) and other specimens. Here, gray filled cells show qualitative PCR (+) for the given miR (columns), offering a miR signature or fingerprint of the airway level.
